# Evaluating Mentors in Violence Prevention: A Longitudinal, Multilevel Assessment of Outcome Changes

**DOI:** 10.1007/s10964-023-01781-y

**Published:** 2023-04-25

**Authors:** Stefania Pagani, Simon C. Hunter, David Lawrence, Mark A. Elliott

**Affiliations:** 1 University of Strathclyde, 16 Richmond Street, Glasgow, G1 1XQ Scotland UK; 2 Glasgow Caledonian University, Cowcaddens Road, Glasgow, G4 0BA Scotland UK; 3 University of Western Australia, 35 Stirling Highway, Perth, WA 6009 Australia; 4 Curtin University, Kent Street, Perth, WA 6102 Australia

**Keywords:** Program evaluation, High school intervention, Gender-based violence, Adolescents, Bystander intervention

## Abstract

There is a need to increase understanding of the effectiveness of bystander programmes targeting gender-based violence in the United Kingdom. There is also a need to utilise a robust theoretical models of decision-making while doing so. Changes were examined in bystanders’ attitudes, beliefs, motivations towards intervening, and intervention behavior in situations of gender-based violence. To achieve this, a quantitative examination of Mentors in Violence Prevention was conducted. There were 1396 participants (50% female, 50% male) who were aged 11 to 14 years old (M = 12.25, SD = 0.84) attending high school at the first time point. Participants were attending 17 schools (53% Mentors in Violence Prevention and 47% control) in Scotland. Outcome variables were assessed approximately one year apart using questionnaires. Multilevel linear regressions revealed that Mentors in Violence Prevention did not change outcomes reflecting bystanders’ attitudes, beliefs, motivations towards intervening, or intervention behavior in gender-based violence. Discrepancies between the current findings and those of other evaluations may be due to other studies including small numbers of schools that may be more motivated to implement the program. This study also identified two key issues that need to be addressed at stakeholder level before concluding that Mentors in Violence Prevention is ineffective at targeting gender-based violence. That the program has moved towards a more gender-neutral approach in the United Kingdom could explain the null results of this study. Furthermore, the current findings could be attributed to a failure to adequately address the theoretical model underpinning the program in practice.

## Introduction

Gender-based violence is violence aimed at someone because of the gender with which they identify (Morrison et al., 2007). It is one of the most common types of violence and it disproportionately targets females (Ellsberg et al., 2020). Research examining gender-based violence can be informed by an understanding of how it develops among adolescents in key social contexts such as schools. Many school programs designed to reduce this violence focus on the role of the bystander (e.g., Amar et al., 2012; Banyard et al., 2007; Cook-Craig et al., 2014; De La Rue et al., 2017; Midgett et al., 2015; Miller et al., 2012). This is because most school-based violence takes place in the presence of others (Polanin et al., 2012). Little is known about the effectiveness of these school-based interventions in the United Kingdom and how they impact on bystanders’ attitudes, beliefs, motivations towards intervening, and intervention behavior when they witness gender-based violence. The current study is the first large-scale, multilevel evaluation of Mentors in Violence Prevention (Katz, 1995), a mentor-led program designed to challenge gender-based violence and violence more generally using a bystander approach, in the United Kingdom. It aimed to examine changes in bystander outcomes that were identified using a robust theoretical model of decision-making (Pagani et al., 2022a).

Within the literature, gender-based violence has been studied in many forms ranging from non-intimate verbal and emotional violence, to dating and relationship abuse, to sexual violence (for recent reviews see De La Rue et al., 2017; Jouriles et al., 2018; Lester et al., 2017; Kettrey & Marx, 2019; Kovalenko et al., 2020). It is important to note that violence itself can manifest in different ways, involving different motivations from different perpetrators which are often complex and difficult to measure. For the purposes of the present study, the term gender-based violence reflects verbal and emotional violence which can involve direct and indirect forms such as name-calling, making fun, spreading rumors, ‘putting down’, and both physical and sexual violence, including pushing, hitting, fighting, sexting, touching, and coercive intercourse (Debnam & Mauer, 2021; Katz et al., 2011; Miller et al., 2012). These forms of violence can occur between boys and girls, both within and outside of romantic relationships. The term gender-based violence also incorporates domestic violence with regards to those in a romantic relationship as the sample explored in this thesis was under the age of 16 (Home Office, 2013).

### Gender-based Violence and a Bystander Approach

Gender-based violence can reflect a motivation to assert power, whether that be within romantic relationships (Debnam & Mauer, 2021), or within peer groups (Skipper & Fox, 2021). This motivation for power can stem from stereotypical attitudes and normative beliefs pertaining to gender identity regarding females as subordinate in society and “lesser” than males (Hindes & Fileborn, 2020; Koss et al., 1994). Schools provide an ideal context in which to examine interactions and relationships between boys and girls because they are the dominant social setting for adolescents. Furthermore, adolescence is an important period in which to examine these relationships as it is a time when gender roles are explored and interpersonal relationships are negotiated (Katz, 2018; Skipper & Fox, 2021).

One way to examine stereotypical attitudes and normative beliefs in gender-based violence contexts is to examine how they manifest and develop in the attitudes, beliefs, and behaviors of those who are present when gender-based violence takes place. Indeed, research has identified that bystanders are present in 85–88% of violent situations in schools (Hawkins et al., 2001). How bystanders react is therefore key to understanding how situations of violence can be challenged since they have the power to facilitate (encourage the perpetrator) or inhibit (help the victim) a violent situation.

There are numerous school-based programs designed to promote bystander intervention in gender-based violence contexts (for meta-analyses and systematic reviews see: DeGue et al., 2014; Jouriles et al., 2018; Lundgren & Amin, 2015; Katz & Moore, 2013; Kettrey & Marx, 2019; Kovalenko et al., 2020; Stanley et al., 2015; Storer et al., 2016). Legislative, policy, and strategic advances have encouraged the implementation of these programs within school contexts. Indeed, the 2013 Campus Sexual Violence Elimination Act (Campus SaVE Act: 2013) made gender-based violence programs in the United States (US) of America mandatory. Most programs have therefore been pioneered and evaluated in the US, with fewer programs and evaluations elsewhere, such as the United Kingdom. Consequently, less is known about the effectiveness of gender-based violence programs in countries outside of the US (Kovalenko et al., 2020).

While the United Kingdom does not have legislation equivalent to the Campus SaVE Act, evaluations into the effectiveness of programs targeting gender-based violence do exist (e.g., Bell & Stanley, 2006; Fox et al., 2020; Hester & Westmarland, 2005; Maxwell et al., 2010; Scottish Executive, 2002; Stanley et al., 2011; Williams & Neville, 2013). However, evidence on the effectiveness of gender-based violence programs that focus on bystanders in secondary schools is scarce within the United Kingdom, with only one peer-reviewed qualitative evaluation existing (Williams & Neville, 2017). Other evaluations exist, but are either not peer-reviewed (e.g., Fox et al., 2020; Fox & Vickers, 2017; Mentors in Violence Prevention Progress Report, 2019; Williams & Neville, 2013) or do not specifically focus on bystander outcomes concerning gender-based violence (Hunter et al., 2021). Methodologically, there is also the need for larger evaluations that include both pre and post intervention testing, control schools, and designs that address the potential for school level differences. These issues are directly addressed in the current study.

### Mentors in Violence Prevention

Mentors in Violence Prevention (Katz, 1995) was originally pioneered in universities in the US. It aims to empower bystanders to challenge negative gendered attitudes, beliefs, and behaviors. The original program was targeted towards male university sports athletes. The remit of Mentors in Violence Prevention has since evolved, and its implementation has expanded to settings such as high schools and involves training both boys and girls as role models. Mentors in Violence Prevention is also now often utilized to tackle violence beyond the original focus on gender-based violence (Katz, 2018). In Scotland, older students in schools (mentors) act as potential role-models for younger students (mentees), and lead younger students through a series of Mentors in Violence Prevention lessons. Mentors in Violence Prevention implements a “train the trainer” approach whereby school staff attend a two-day training course that is run by development officers. Once they have received this initial training, school staff assume the position of Mentors in Violence Prevention leads and go on to train mentors (schools students) to implement lessons, utilising the same training that they themselves received (Mentors in Violence Prevention Progress Report, 2016). For each lesson, there is a lesson plan with a clear structure for mentors to follow. Lessons are usually incorporated into a normal school lesson with mixed gender classes (the size and structure depending on that of the school in question), replacing subjects such as Social Education. Lessons involve leading younger students through a hypothetical scenario which depicts a situation of gender-based violence or violence more generally. Situations include verbal, emotional, physical, and/or sexual violence. Following the presentation of the scenario, mentors engage the younger students in a “train of thought” about the scenario, allowing them to consider their beliefs, attitudes, and thought patterns. Finally, seven possible bystander reactions are presented, and they are invited to discuss the positive and negative consequences of each one. Mentors in Violence Prevention has five core values which mentors are encouraged to cover in each of their lessons: violence through a gendered lens, using a bystander approach, developing leadership, recognising the scope of violence, and challenging victim blaming.

Mentors in Violence Prevention has been implemented in the United Kingdom since 2012, first in Scotland and then in England in 2015. Both university and high school evaluations have been conducted in the US (Beardall, 2007; Cissner, 2009; Eriksen, 2015; Heisterkamp et al., 2011; Katz et al., 2011; Ward, 2001) and UK (Fox et al., 2020; Fox & Vickers, 2017; Hunter et al., 2021; Mentors in Violence Prevention Progress Report, 2019; Williams & Neville, 2013, 2017) respectively. Positive effects have been reported in relation to attitudes towards intervening (Beardall, 2007; Cissner, 2009; Eriksen, 2015; Fox et al., 2020; Heisterkamp et al., 2011; Katz et al., 2011; Mentors in Violence Prevention Progress Report, 2019; Ward, 2001; Williams & Neville, 2013, 2017), self-efficacy towards intervening (Beardall, 2007; Cissner, 2009; Eriksen, 2015; Fox & Vickers, 2017; Ward, 2001; Williams & Neville, 2017), and intentions to intervene (Cissner, 2009; Eriksen, 2015; Heisterkamp et al., 2011; Katz et al., 2011; Mentors in Violence Prevention Progress Report, 2019; Ward, 2001; Williams & Neville, 2017). Two studies also noted overall decreases in school rates of gender-based violence after the intervention (Cissner, 2009; Heisterkamp et al., 2011). However, not all studies have included control schools (Beardall, 2007; Eriksen, 2015; Fox et al., 2020; Fox & Vickers, 2017; Mentors in Violence Prevention Progress Report, 2019; Williams & Neville 2017), or pre and post testing methods (Beardall, 2007; Fox & Vickers, 2017; Hunter et al., 2021; Katz et al., 2011; Mentors in Violence Prevention Progress Report, 2019; Williams & Neville, 2017), which are important in order to draw reliable comparisons (between similar schools or the same school at different time points) that provide a more robust and accurate insight into the effectiveness of an intervention (e.g., Kovalenko et al., 2020). Many studies also included small numbers (two or three) of intervention schools (Beardall, 2007; Cissner, 2009; Eriksen, 2015; Heisterkamp et al., 2011; Katz et al., 2011; Williams & Neville, 2013, 2017). Some of these studies that did not use control or pre and post testing methods, and included a small number of schools, found strong, positive Mentors in Violence Prevention effects or perceived effects (Beardall, 2007; Katz et al., 2011; Williams & Neville, 2013), whereas others found more mixed or even null effects (Eriksen, 2015; Fox et al., 2020; Hunter et al., 2021; Ward, 2001; Williams & Neville, 2013). Some of these latter studies also examined larger numbers of schools (Fox et al., 2020; Hunter et al., 2021; Ward, 2001). Nonetheless, other studies that have used control schools, did find positive intervention effects (Cissner, 2009; Heisterkamp et al., 2011), however, these studies included a small number of schools: two and four respectively.

Studies have examined the effectiveness of bystander intervention programs by focussing on the individual bystander and therefore changes in individual social cognitive factors that are influential when bystanders are making the decision over whether or not to intervene (e.g., Debnam & Mauer, 2021; Hoxmeier et al., 2018; Sjögren et al., 2021; Thornberg & Wänström, 2018). These factors are components of theoretical models of decision-making, such as the Theory of Planned Behavior (Ajzen, 1988, 1991) and the Prototype Willingness Model (Gibbons & Gerrard, 1995, 1997). Unfortunately, many of these studies have only examined some of the factors that comprise these theoretical models, leading to a partial picture of bystander decision-making. There is therefore a need for more studies to examine all factors that comprise theoretical models of bystander decision-making (Pagani et al., 2022a). Similarly, there is also a need for studies to assess changes in all these factors.

This Prototype Willingness Model has been tested to identify the predictors of bystander intervention behavior in gender-based violence contexts (Pagani et al., 2022a). Those constructs that were found to successfully predict bystander intervention were: willingness to intervene in less serious gender-based violence; positive attitudes (positive evaluations of intervening); negative attitudes (negative evaluations of intervening); subjective norms (beliefs about other bystanders intervening); self-efficacy (perceived ability to intervene); prototype perceptions (identification with the typical bystander who intervenes); moral disengagement (beliefs about whether intervening is the right thing to do). Some of these factors (positive and negative attitudes towards intervening, prototype perceptions concerning self-comparison to the typical bystander who intervenes regularly, willingness to intervene) have been novelly tested in the context of bystander decision-making (Pagani et al., 2022a). However, these factors have been shown to predict decision-making in a range of other situations, including speeding, binge-drinking, smoking, and consuming a high-fat diet (Elliott et al., 2015; McCartan et al., 2018). Other factors (subjective norms concerning beliefs about other bystanders’ intervention behaviors, self-efficacy concerning confidence in one’s own ability to intervene, and moral disengagement by justifying gender-based violence) have also been extensively examined in the field of bystander decision-making (e.g., Rosval, 2013, Sjögren et al., 2021; Thornberg & Jungert, 2014). These factors have been examined within other theoretical models of bystander decision-making, such as the Theory of Planned Behavior (Ajzen, 1991).

The constructs that did not significantly predict bystander intervention behavior (Pagani et al. 2022a) were: intentions (likelihood to intervene); willingness to intervene in more serious gender-based violence; and perceived behavioral control (belief about whether intervening is under one’s own control). However, willingness predicts behavior in a range of other situations (e.g., Elliott et al., 2015). Like subjective norms, perceived behavioral control is a construct of the Theory of Planned Behavior, however, it has been replaced or conflated with self-efficacy in the bystander literature (Rosval, 2013; Salmivalli, 2010; Sjögren et al., 2021). However, factor analytic studies have illustrated their independent effects in predicting intentions and behavior (Armitage & Conner, 1999a, 1999b). The predictive ability of intentions has been examined widely in different bystander situations (e.g., Leone & Parrott, 2019; McMahon et al., 2015; Rosval, 2013). Therefore, the inclusion of these factors would ensure a theoretically comprehensive range of social cognitions found to predict many other health-related behaviors (Elliott et al., 2015; Hoxmeier et al., 2018; McCartan et al., 2018; McCartan & Elliott, 2018; Rosval, 2013).

## Current Study

The present study aimed to address the above stated limitations by examining changes in bystanders’ attitudes, beliefs, motivations towards intervening, and intervention behavior after having received the Mentors in Violence Prevention intervention, and by using outcomes that comprise a robust theoretical model of decision-making. To achieve this, both control and pre and post testing methods were used, with a 12-month follow-up to help provide a better insight into the mixed findings above in relation to the different methods used. In addition, effectiveness was tested using both measures of bystander intervention behavior and a comprehensive range of socio-cognitive constructs that comprise a robust theoretical model of decision-making. Specifically, this study used an augmented Prototype Willingness model. Those decision-making constructs above that were found to significantly predict bystander intervention behavior were deemed the most suitable outcome variables (due to their significant influence in bystander decision-making using the same sample as used in this study) for assessing the effectiveness of Mentors in Violence Prevention. They were therefore included in the confirmatory analyses in this study. In line with the preregistration^1^ for this study on the Open Science Framework (OSF: 10.17605/OSF.IO/ZCJS6), it was therefore hypothesized that: compared to those in non-intervention schools, students in Mentors in Violence Prevention schools would have significantly larger changes in these outcome variables and both positive intervention behavior (e.g., confronting the perpetrator of gender-based violence) and negative intervention behavior (e.g., doing nothing). The constructs above that did not significantly predict bystander intervention behavior were included in exploratory analyses in the current study.

## Method

### Participants

The final sample at both T1 and T2 was 1396 (50% attending Mentors in Violence Prevention (MVP) and 50% attending non-MVP schools) students attending 17 (9 MVP and 8 non-MVP) mainstream high schools in Scotland between Autumn 2018 and Spring 2020. Participants were aged 11 to 14 years old (M = 12.25, SD = 0.84). Overall, 689 (49.4%) reported their gender as female, 685 (49.1%) as male, 9 (0.6%) “prefer not to say”, and 13 (0.9%) did not report. Ninety one percent of the sample identified as “White Scottish or White British” (*N* = 1270), 2.4% identified as “Asian, Asian Scottish/ British”, 0.9% identified as “African”, 1.9% identified as “Mixed or multiple ethnic group”, 0.4% identified as “Caribbean or Black” and 2.9% identified as “Other ethnic group”. Socioeconomic status, as assessed by the percentage of students in each school who were registered for free school meals, ranged from 1.0% - 50.2% (M = 20.6%, SD = 14.0).

In the preregistration (OSF: 10.17605/OSF.IO/ZCJS6), simulation analyses were conducted (Castelloe and O’Brien, 2001) to determine if the sample size was sufficient for the anticipated design of the study, that is, a multilevel, longitudinal design. The same sample recruited was used here as in another study (Pagani et al., 2022a), though the current study includes data from a second time point which occurred approximately 12 months after the first. T1 data therefore informed the simulation approach, which indicated that the sample sizes across anticipated outcomes were sufficient to detect meaningful effect sizes (small-medium effects *d* = 0.25–0.35; Cohen, 1992) across all the outcomes. However, the anticipated sample size (N = 671) for the more serious intervention behavior outcomes was considerably more than the observed sample size (N = 288).^2^

### Design and Procedure

An observational, longitudinal design was used, where schools were pre-allocated to MVP and non-MVP groups. All anticipated outcome variables were measured at T1 (between Autumn 2018 and Spring 2019), and then subsequently again at T2, approximately one year later. Covariates (gender, age, ethnicity, empathy) were measured at T1 only.

A total of 2079 young people in S1-S3 attending 19 (14 MVP, 5 non-MVP) mainstream secondary schools in Scotland consented to take part at T1. However, between T1 and T2, five of the ‘MVP’ schools were unable to implement MVP due to staffing and structural issues: therefore, three subsequently became non-MVP schools and two withdrew entirely due to school closures related to the COVID-19 pandemic. In addition, and as a result of the pandemic, two more non-MVP schools had completed partial data collection before they were forced to close. Consequently, one school reported on intervention behavior only at T2 and the other school reported on all other variables only at T2. Both were included in the analyses. As described above, the final sample for T1 and T2 was 1396 students, with 50% (698) attending non-MVP schools and 50% attending MVP schools. However, participation within schools varied considerably due to whether positive or negative consent was sought as well as the availability of the target age groups at the time of fieldwork. Numbers of participants varied from 5 to 198 between schools.

Ethical approval for this study was granted by the lead author’s institution. Scottish Local Education Authorities (LEAs) were contacted for permission to approach schools. Permission was obtained from 10 of 13 (77%) LEAs. All schools within the ten authorities were invited to take part. Of the schools contacted, 42 (26%) expressed interest in taking part and 19 did so. Time, resource limitations, and eligibility prevented some schools from participating. The research team aimed to recruit a balanced number of MVP and non-MVP schools. However, the fact that more MVP schools (14) opted into the study than non-MVP schools (5) potentially reflected the commitment of these schools to the MVP program or indeed their motivation to understand the effectiveness of a program that they had committed so much time and resource to. Schools could not be randomly assigned to these groups as the implementation of MVP is led by the Scottish Government and thus randomization was not within the control of the research team.

Information letters and consent forms were distributed to all parents whose children were eligible. At the preference of the LEA, either negative (80% of LEAs) or positive (20%) consent was sought. Parents were given at least one week to return consent. Each young person also consented before participation. Participants completed anonymous questionnaires within a classroom or assembly hall context. At both T1 and T2, data collection occurred in two phases: all measures (see section below) except intervention behavior were completed in the first phase and reported intervention behavior was assessed in a second phase approximately one month later. The questionnaire completed in phase 1 took one school period (45–55 min) to complete, and 5–10 min to complete in phase 2. Teachers and members of the research team were present during data collection. Participants were subsequently debriefed. Students who were not participating were usually given another classroom task to complete or attended a non-participating classroom that was undertaking their work as normal.

### Measures

Scale items were drawn from the literature (e.g., Miller et al., 2012) for each measure. Eight gender-based violence examples (Miller et al., 2012; see Appendix 1 for a description of the eight examples, divided into their verbal/emotional and physical/sexual counterparts) were integrated within each measure. These included examples of emotional, verbal, physical, and/or sexual violence. All eight gender-based violence examples were not used to assess every construct to reduce burden, but examples were included so that a balanced range of verbal/emotional and physical/sexual violence items assessed each. For most measures (positive and negative attitudes towards intervening, self-efficacy, perceived behavioral control over intervening, subjective norms concerning beliefs about other bystanders’ intervention behaviors, prototype perceptions concerning self-comparison to the typical bystander who intervenes regularly, willingness to intervene), questions were split into parts “a” and “b”, where part “a” reflected an example of verbal/emotional violence and part “b” reflected an example of physical/sexual violence. For measures that were structured differently (intentions to intervene, self-reported behavior, moral disengagement by justifying gender-based violence), a balanced mix of verbal/emotional (4 items for intentions and self-reported behavior, and 3 items for moral disengagement) and physical/sexual (4 items for intentions and self-reported behavior, and 3 for moral disengagement) examples were incorporated into the questions. Participants were thus exposed equally to the eight examples. The only measure that did not take this approach was empathy and this was because it measured a personality trait. All outcome variables were measured using unipolar scales in line with standard practice (e.g., Fishbein & Ajzen, 2010). The factorial structures of the measures follow that in another study (Pagani et al., 2022a) and so, factor scores were created for each measure by summing each item’s raw scores multiplied by that item’s factor weight.

### Outcome variables for confirmatory analyses

#### Attitudes towards intervening

Both positive and negative attitudes were measured separately (Elliott et al., 2015). Six items were used to measure positive attitudes towards intervening (e.g., How positive would it be if you did something about it if you saw [*violence example*]). The participants were asked to respond to each item using a 9-point unipolar scale (e.g., 1 = *not at all positiv*e to 9 = *extremely positive*). Similarly, six items were used to measure negative attitudes towards intervening (e.g., How negative would it be if you did something about it if you saw [*violence example*]). The participants were asked to respond to each item using a 9-point unipolar scale (e.g., 1 = *not at all negativ*e to 9 = *extremely negative*). For both the positive and negative scales, each of the six items were divided into “a” and “b” components, essentially making each of the scales consist of 12 indices. Factor scores were created where a higher score indicated higher positive or higher negative attitudes. Internal reliability was high at T1 (positive scale α = 0.93; negative scale α = 0.93) and T2 (positive scale α = 0.93; negative scale α = 0.92).

#### Subjective norms concerning beliefs about other bystanders intervening

A three-item scale was adapted (Wilson et al., 2016). Participants rated responses to questions like, “Of the students you know, how many do you think will do something about it over the next month when they see…(*violence example*)”, from 1 = *none of them* to 9 = *all of them*. For this scale, each of the three items were divided into “a” and “b” components, essentially making the scale consist of six items. Factor scores were created, and a higher score indicated higher levels of perceived social pressure to intervene. Internal reliability was high at T1 (α = 0.82) and T2 (α = 0.79).

#### Self-efficacy concerning confidence in one’s own ability to intervene

A three-item scale was adapted (Wilson et al., 2016). Participants rated responses to questions like, “Over the next month, I have the ability to do something about it when I see… (*violence example*)”, from 1 = *not at all confident* to 9 = *very confident*. For this scale, each of the three items were divided into “a” and “b” components, essentially making the scale consist of six items. Factor scores were generated where a higher score indicated higher self-efficacy to intervene. Internal reliability was high at T1 (α = 0.75) and T2 (α = 0.73).

#### Prototype perceptions concerning self-comparison to the typical bystander who intervenes regularly

A four-item scale was adapted (Elliott et al., 2017). Participants rated responses to questions like “Do you resemble the type of person your age that regularly does something about it when they see… (*violence example*)”, from 1 = *definitely no* to 9 = *definitely yes*. For this scale, each of the four items were divided into “a” and “b” components, essentially making the scale consist of eight items. Factor scores were created, and higher scores indicated that participants regarded themselves as being more like the perceived prototypical student who intervenes regularly. Internal reliability was high at T1 (α = 0.91) and T2 (α = 0.90).

#### Moral disengagement concerning justification of gender-based violence

A six-item scale was adapted (Thornberg & Jungert, 2014). Participants rated responses to questions like, “It’s okay for… (*violence example*)”, from 1 = *strongly agree* to 7 = *strongly disagree*. As described at the start of this section, three of the items included examples of verbal/emotional violence and the other three included examples of physical/sexual violence. Factor scores were created, and items were reverse scored so that a higher score indicated higher moral disengagement. Internal reliability was high at T1 (α = 0.91) and T2 (α = 0.90).

#### Behavioral willingness to intervene

A three-item scale was adapted (Elliott et al., 2017). Participants rated responses to questions like, “Suppose you saw…(*violence example*)…over the next month and no-one else there was doing anything about it/ none of your friends were intervening/ no-one else was around. How willing would you be to do something?” (1 = *not at all willing* to 9 = *extremely willing*). For this scale, each of the three items were divided into “a” and “b” components, essentially making the scale consist of six items. The factor analytic approach revealed that this measure constituted “less serious” and “more serious” gender-based violence components, where “a” items loaded onto one factor and “b” items loaded onto another. These were therefore included as separate measures. Internal reliability was high for T1 (willingness_MoreSerious_: α = 0.82; willingness_LessSerious_: α = 0.86), and T2 (willingness_MoreSerious_: α = 0.88; willingness_LessSerious_: α = 0.89).

#### Self-reported intervention behavior

Mentees reported if they had witnessed eight gender-based violence situations in the previous month (Miller et al., 2012). For each situation witnessed, participants then reported how they had intervened by ticking box(es) reflecting two negative (e.g., “I didn’t do/say anything”), and four positive (e.g., “I told the person in public that acting like that was not ok”) responses (Miller et al., 2012). As described at the start of this section, four of the items included examples of verbal/emotional violence and the other four included examples of physical/sexual violence. This measure also contained more serious and less serious components of gender-based violence (Pagani et al., 2022a), resulting in four measures of intervention behavior: the proportion of times the mentees intervened positively in less serious situations (positive intervention_LessSerious_), that which they intervened positively in more serious situations (positive intervention_MoreSerious_), that which they intervened negatively in less serious situations (negative intervention_LessSerious_) and that which they intervened negatively in more serious situations (negative intervention_MoreSerious_).

There were 1136 mentees who responded to the self-reported bystander behavior questionnaire at both T1 and T2. This is slightly smaller than the reported number of participants (n = 1396) and could likely reflect student absences as well as refusals to complete the questionnaires on the date that this fieldwork was completed, which was around one month after the longer questionnaire, as described in the earlier design and procedure. Table 1 shows the proportions of youth who reported witnessing the eight possible gender-based violence situations examined in this report. These are split by both time points and by MVP vs. non-MVP schools.

**Table 1:** Proportions of participants who had witnessed between 0 and 4 situations of more and less serious gender-based violence at T1 and T2 in MVP, non-MVP, and overall groups

		More serious gender-based violence			Less serious gender-basedviolence			
		No opportunity	Saw 1–2 situations	Saw 3–4 situations	No opportunity	Saw 1–2 situations	Saw 3–4 situations	Count
MVP	T1	60.28%	33.51%	6.21%	46.46%	41.11%	12.43%	557
	T2	57.86%	35.23%	6.91%	34.02%	49.91%	16.07%	
NON-MVP	T1	54.58%	38.06%	7.36%	26.57%	44.70%	28.73%	579
	T2	53.68%	36.80%	9.52%	20.83%	46.32%	32.85%	
OVERALL	T1	57.50%	35.74%	6.76%	36.71%	42.87%	20.42%	1136
	T2	55.81%	36.00%	8.19%	27.55%	48.15%	24.30%	

These proportions only reflect whether or not bystanders had the opportunity to intervene in each of the eight situations of gender-based violence and not the rates of opportunities they had within each of the situations. Exact numbers are not reported on as some of the data did not contain numerical estimates, but instead descriptive text that reflected the amount of times they had witnessed each situation, for example, “lots” or “too many to count”.

### Covariates

#### Gender

Gender was reported as “a boy”, “a girl”, or “prefer not to say”. Those who responded “prefer not to say” or with missing responses were omitted due to small numbers (1.5% of total responses). Gender was coded as 0 = *boy* and 1 = *girl* for analyses.

#### Age

Ages ranged from 11–15. No treatment of this variable occurred for analyses.

#### Ethnicity

Participants reported their ethnicity as “White Scottish or White British”, “Asian, Asian Scottish/ British”, “African”, “Mixed or multiple ethnic group”, “Caribbean or Black”, and “Other ethnic group”. Due to small numbers in each of these categories of response aside from “White Scottish or White British” (see participants section), all other ethnic groups were collapsed into one group “other ethnic group”. Ethnicity was coded as 0 = *White Scottish or White British*, and 1 = *other ethnic group* for analyses.

#### Empathy

A six-item scale was used (Caravita et al., 2009), where participants rated responses to questions like, “Seeing a friend crying makes me feel as if I am crying”, from 1 = *never true* to 4 = *always true*. A mean score was created where a higher score indicated higher affective empathy. Internal reliability was satisfactory (α = 0.77).

### Outcome variables for exploratory analyses

#### Perceived behavioral control concerning perceptions of one’s control over intervening

A two-item scale was adapted (Wilson et al., 2016). Participants rated responses to questions like, “Over the next month, how much personal control do you feel you have over doing something about it when you see… (*violence example*)”, from 1 = *no control at all* to 9 = *complete control*. For this scale, each of the two items were divided into “a” and “b” components, essentially making the scale consist of four items. Factor scores were created, and a higher score indicated higher perceived control over intervening. Internal reliability was high at T1 (α = 0.88) and T2 (α = 0.90).

#### Intentions to intervene

An eight-item scale was adapted (Miller et al., 2012). Participants rated responses to questions like “How likely are you to do something about it over the next month if a male peer / friend is…(*violence example*)”, from 1 = *very unlikely* to 5 = *very likely*. As described at the start of this section, four of the items included examples of verbal/emotional violence and the other four included examples of physical/sexual violence. Factor scores were generated where a higher score indicated higher intentions to intervene. Internal reliability for this scale was high at T1 (α = 0.95) and T2 (α = 0.93).

### Analytic Plan

A series of confirmatory multilevel linear regressions were planned for the anticipated outcomes. A multiple indicator factor-analytic approach was preregistered to be used to compute latent change scores representing outcome variables. However, the number of parameter estimates (116) outnumbered the number of clusters (16 schools) in the study, and so a more parsimonious model was required. An observed change score was thus computed (Castro-Schilo & Grimm, 2018) for each outcome variable by subtracting factor scores at T1 from those at T2. The predictor was MVP (0 = *non-MVP*, 1 = *MVP*), and the covariates were gender, age, ethnicity, and affective empathy. The multilevel approach allowed within school effects to be estimated using intraclass correlations. Three exploratory multilevel linear regressions were also planned. Due to the large number of analyses, alpha for exploratory analyses was adjusted to *p* < 0.01.

## Results

### Treatment of Missing Data

Participants with any partial missing data across the outcome variables were deleted, therefore, final sample sizes within each analysis varied. With the exception of the self-reported intervention behavior outcome variables, data were missing completely at random, and sample sizes varied from 1075 (23.0% missingness) to 1223 participants (12.4% missingness). For the self-reported intervention behavior outcome variables, missing data was determined by whether or not participants had the opportunity to intervene in more serious or less serious gender-based violence in the preceding month (see measures section). This was true for both T1 and T2, so some participants who had the opportunity to intervene at T1 may not have had the opportunity at T2 and vice-versa. For the more serious intervention behavior outcome variables, the sample size was 288 (79.4% missingness) who had the opportunity to intervene at both T1 and T2. For the less serious intervention behavior outcome variables, the sample size was 618 (55.5% missingness) who had the opportunity to intervene at both T1 and T2. For the covariates, a Full Information Maximum Likelihood (FIML) approach was implemented in MPlus to address any missing data.

### Descriptive Statistics

Table 2 shows the mean change scores and intraclass correlation (ICC) coefficients for all outcome variables, representing those for MVP and non-MVP schools separately as well as those for MVP and non-MVP schools combined.

**Table 2:** Mean change score and ICCs for confirmatory outcomes across schools

	MVP Schools Change Scores		Non-MVP Schools Change Scores		Overall Change Scores	
	Mean	ICC	Mean	ICC	Mean	ICC
positive attitudes^+^	0.05	0.00	−0.04	0.01	0.00	0.01
negative attitudes^-^	−0.16	NC	−0.07	0.00	−0.11	0.01
subjective norms^+^	−0.02	0.00	−0.02	0.02	−0.02	0.01
self-efficacy^+^	−0.02	0.00	−0.01	NC	−0.01	0.00
prototype perceptions^+^	−0.03	0.02	−0.00	NC	−0.01	0.02
moral disengagement^_^	−0.11	NC	−0.06	NC	−0.08	0.00
willingness_LessSerious_^+^	−0.06	0.00	−0.04	0.03	−0.05	0.01
positive intervention_LessSerious_^+^	0.02	NC	0.03	0.06	0.02	0.02
positive intervention_MoreSerious_^+^	−0.09	NC	0.05	0.09	−0.02	0.05
negative intervention_LessSerious_^-^	−0.04	0.01	−0.04	NC	−0.04	0.00
negative intervention_MoreSerious_^-^	0.01	0.08	−0.02	0.04	−0.01	0.05

*NC* Non computable (can occur when ICCs are extremely small), *ICC* intraclass correlation coefficient, willingness_LessSerious_ = willingness to intervene in less serious gender-based violence; positive intervention_LessSerious_ = positive intervention in less serious gender-based violence; positive intervention_MoreSerious_ = positive intervention in more serious gender-based violence; negative intervention_LessSerious_ = negative intervention in less serious gender-based violence; negative intervention_MoreSerious_ = negative intervention in more serious gender-based violence. ^+^ = if the mean change score is positive then this indicates an improvement from T1 to T1. ^-^ = if the mean score is negative then this indicates an improvement from T1 to T2

The differences between T1 and T2 scores across all outcomes were not statistically significant for non-MVP schools. For MVP schools, negative attitudes towards intervening, t (df = 565) = 2.91, *p* = 0.002, d = −0.12, and moral disengagement by justifying gender-based violence, t (df = 548) = 2.55, *p* = 0.006, d = −0.11, improved from T1 to T2. However, positive intervention_MoreSerious_ (positive intervention in more serious gender-based violence) deteriorated from T1 to T2, t (df = 135) = 1.85, *p* = 0.033, d = −0.16.

ICC scores ranged from 0.00 to 0.09 (Median = 0.01) in non-MVP schools and 0.00–0.08 (Median = 0.08) in MVP schools, indicating that some outcomes (e.g., prototype perceptions, negative_MoreSerious_) had higher similarity among those within the same schools than others (e.g., negative attitudes towards intervening, self-efficacy concerning confidence in one’s own ability to intervene), and that non-MVP schools generally had more outcomes showing higher similarity between those in the same school than MVP schools.

### Confirmatory Regression Analyses

Tables 3 and 4 present the results of the confirmatory regression analyses testing the effects of MVP on the change scores for the outcome variables.

**Table 3:** Unstandardized estimated effects on change scores for outcome variables, including within and between effects

	positive attitudes		negative attitudes		self-efficacy		subjective norms		prototype perceptions		moral disengagement	
	B	*p*-value	B	*p*-value	B	*p*-value	B	*p*-value	B	*p*-value	B	*p*-value
*Within level*												
Gender	0.06	0.498	−0.06	0.175	−0.01	0.850	−0.08	0.142	0.00	0.994	−0.02	0.778
Ethnicity	0.24	0.020	0.05	0.694	0.02	0.643	0.03	0.564	−0.10	0.676	−0.01	0.944
Empathy	−0.07	0.455	0.02	0.812	0.07	0.025	−0.04	0.376	−0.02	0.776	−0.01	0.919
Age	−0.01	0.793	−0.07	0.140	0.02	0.356	−0.03	0.041	0.08	0.268	0.03	0.607
*Between level*												
MVP intervention	0.10	0.112	−0.03	0.556	−0.00	0.997	−0.01	0.969	0.03	0.827	−0.10	0.170

**Table 4:** Unstandardized estimated effects on change scores for outcome variables, including within and between effects

	Willingness_LessSerious_		Positive intervention_MoreSerious_		Positive intervention_LessSerious_		Negative intervention_MoreSerious_		Negative intervention_LessSerious_	
	B	*p*-value	B	*p*-value	B	*p*-value	B	*p*-value	B	*p*-value
*Within level*										
Gender	0.03	0.646	0.05	0.627	0.05	0.095	0.03	0.668	0.03	0.429
Ethnicity	−0.11	0.074	0.14	0.349	0.00	0.958	0.03	0.814	−0.06	0.435
Empathy	0.01	0.838	0.00	0.967	0.04	0.327	−0.02	0.822	−0.07	0.328
Age	−0.02	0.711	−0.03	0.783	0.07	0.012	−0.07	0.256	−0.06	0.001
*Between level*										
MVP intervention	−0.07	0.646	−0.03	0.796	0.02	0.674	0.03	0.645	−0.02	0.625

willingness_LessSerious_ = willingness to intervene in less serious gender-based violence; positive intervention_LessSerious_ = positive intervention in less serious gender-based violence; positive intervention_MoreSerious_ = positive intervention in more serious gender-based violence; negative intervention_LessSerious_ = negative intervention in less serious gender-based violence; negative intervention_MoreSerious_ = negative intervention in more serious gender-based violence

As can be seen in Tables 3 and 4, the hypotheses were not supported: MVP did not significantly predict any change score. Regarding covariates, gender had no significant effect. Ethnicity significantly predicted change scores in positive attitudes, where those who are not “White Scottish/White British” had larger improvements in positive attitudes towards intervening in gender-based violence. Age significantly predicted change scores in subjective norms (beliefs about whether others would intervene), negative intervention_LessSerious_ (negative intervention in less serious violence), and positive intervention_LessSerious_ (positive intervention in less serious violence). Specifically, as age increased the change scores in subjective norms concerning beliefs about other bystander’ intervention behaviors deteriorated, and the change scores in positive and negative intervention_LessSerious_ improved. Empathy also predicted self-efficacy, so as empathy increased, self-efficacy concerning confidence in one’s own ability to intervene improved.

### Exploratory Regression Analyses

Perceived behavioral control over intervening increased significantly from T1 to T2 in both MVP, t (df = 553) = −3.63, *p* < 0.001, d = 0.15, and non-MVP, t (df = 596) = −3.46, *p* < 0.001, d = 0.14, schools. ICC scores were 0.01 for MVP schools and 0.00 for non-MVP schools. MVP exposure did not have an effect on this variable’s change score (B = 0.03, *p* = 0.737), suggesting that those in MVP schools did not change significantly in their levels of perceived behavioral control over intervening compared to those in non-MVP schools. There was also no effect for the covariates.

Behavioral intentions to intervene increased significantly from T1 to T2 in both MVP, t (df = 547) = −4.56, *p* < 0.001, d = 0.20, and non-MVP, t (df = 611) = −2.89, *p* = 0.002, d = 0.12, schools. It was not possible to compute ICC scores (potentially because they were extremely small) for MVP schools though this was 0.01 for non-MVP schools. MVP exposure did not have an effect on this variable’s change score (B = 0.10, *p* = 0.135), however gender (B = −0.21, *p* < 0.001) and empathy (B = 0.11, *p* = 0.007) did. This suggests that those in MVP schools did not change significantly in intentions compared to those in non-MVP schools, that intentions to intervene improved more for boys and that, as empathy increased, intentions to intervene improved more.

Willingness_MoreSerious_ to intervene did not change significantly from T1 to T2 in MVP (Mean_Change_ = −0.01, *p* = 0.424), but increased in non-MVP schools, t (df = 572) = −1.79, *p* = 0.033, d = 0.10, schools. ICC scores were 0.03 in MVP schools and 0.02 in non-MVP schools. MVP exposure did not have an effect on the change score for this variable (B = 0.03, *p* = 0.737), suggesting that those in MVP schools did not change significantly in their levels of willingness_MoreSerious_ compared to students in non-MVP schools. There were also no effects for the covariates.

## Discussion

There are few peer-reviewed studies that contain pre- and post-intervention and control methods to examine the effectiveness of programmes aimed at tackling gender-based violence by using a bystander intervention approach in the United Kingdom. Furthermore, existing studies, including those that are conducted outside of the United Kingdom, tend to provide a partial examination of bystander decision-making factors, rather than a full set of factors that comprise a robust theoretical model of decision-making. This study aimed to address these issues by providing insight into changes in bystander attitudes, beliefs, motivations, and behaviors when violence is witnessed among Scottish high school students. These outcomes were identified using a robust theoretical model of decision-making (Pagani et al., 2022a) which informed the Mentors in Violence Prevention intervention program. This intervention takes a mentor-led approach to target bystanders’ attitudes, beliefs, motivations towards intervening, and intervention behavior in different situations of gender-based violence. This study was the first large-scale multilevel evaluation in the United Kingdom. Intervention exposure did not significantly impact on anticipated outcomes, including positive and negative attitudes towards intervening, self-efficacy concerning confidence in one’s own ability to intervene, subjective norms concerning beliefs in other bystanders’ intervention behaviors, prototype perceptions concerning self-comparison to the typical bystander who intervenes regularly, positive intervention behavior, or negative intervention behavior. Neither were there between school variations in the effect of the intervention. Exploratory analyses revealed that Mentors in Violence Prevention did not have an effect on perceived behavioral control over one’s own intervention behavior, intentions to intervene, or willingness to intervene in more serious gender-based violence. The results do not therefore provide any evidence that Mentors in Violence Prevention was effective at modifying the anticipated outcomes pertaining to bystander decision-making and intervention in different situations of gender-based violence.

These findings are in line with other research finding no evidence for the effectiveness of Mentors in Violence Prevention (Hunter et al., 2021). Nevertheless, it should be noted that the majority of relevant evaluation research has found some positive effects for the program. Specifically, and in direct contrast to this study, significant differences in self-efficacy concerning confidence in one’s own ability to intervene (Beardall, 2007; Cissner, 2009; Eriksen, 2015; Fox & Vickers, 2017; Ward, 2001; Williams & Neville, 2017), and intentions to intervene (Cissner, 2009; Eriksen, 2015; Heisterkamp et al., 2011; Katz et al., 2011; MVP Progress Report, 2019; Ward, 2001; Williams & Neville, 2017) have been reported.

Methodological differences between the current study and Mentors in Violence Prevention evaluations reporting positive effects may partially explain the discrepancies between the results. This study included a large number of schools from multiple Local Education Authorities across Scotland. Many of the other evaluations examined the effectiveness of Mentors in Violence Prevention in only one (Beardall, 2007; Cissner, 2009; Eriksen, 2015), two (Katz et al., 2011), or three schools (Heisterkamp et al., 2011; Williams & Neville, 2013, 2017). Indeed, studies that evaluated intervention effectiveness across a larger number of schools tended to yield more mixed or null findings (Fox et al., 2020; Hunter et al., 2021; Ward, 2001). Thus, it is possible that studies involving only a very small number of schools may also have recruited very committed schools who are more effective at producing change as a result.

Cultural variations may also help to explain the differences in findings between the evaluations conducted in America and England and those conducted in Scotland. For example, the Mentors in Violence Prevention program was adapted specifically for a Scottish context (Mentors in Violence Prevention Progress Report, 2016) by referring to knife crime rather than gun crime, which may mean that implementation in Scotland differs from that in America. Although, adaptations to suit the cultural context should be beneficial (Durlak & DuPre, 2008). Indeed, Mentors in Violence Prevention leads in schools are encouraged to focus on violent situations in lessons that are relevant to the school’s own culture. This could potentially mean that leads choose to focus on very specific situations of violence, that may not include gender-based violence specifically or may not fully cover all the examples of gender-based violence that they were asked about in this study. Furthermore, when developing the research questions for this study, the gender-based violence situations that were included aligned to those discussed within Mentors in Violence Prevention lessons specifically.

Another potential explanation to consider for the null findings is implementation variation (DeGue et al., 2014; Durlak & DuPre, 2008; Jouriles et al., 2018; Kovalenko et al., 2020; Storer et al., 2016). However, published work utilising the same sample used in this article (Pagani et al., 2022b) examining the implementation of Mentors in Violence Prevention, found no dosage (level of exposure to the program), fidelity (extent to which core components such as gender-based violence and bystander intervention were covered during lessons), or adaptation (extent to which components of the program were adapted) effects. However, the change of Mentors in Violence Prevention’s scope to also focus on more general violence (Katz, 2018) could potentially dilute the communication of gender-based violence, essentially making it a tick-box exercise (Pagani et al., 2022b), given that mentors are encouraged to cover this as a core value of the intervention. This could be another example of cultural differences across Scotland and America, where the evaluations conducted in America may have been conducted within schools which were fully supportive of the primary focus being on gender-based violence. Evidence has also emerged that some schools in England have not directly addressed gender-based violence at all when implementing Mentors in Violence Prevention (Fox et al., 2020).

Systematic reviews and meta-analyses often report mixed effects, with a large number of programs having no effects (e.g., DeGue et al., 2014; Jouriles et al., 2018; Lundgren & Amin, 2015; Katz & Moore, 2013; Kettrey & Marx, 2019; Kovalenko et al., 2020; Stanley et al., 2015; Storer et al., 2016). A common explanation put forward here is that many gender-based violence programs lack a strong theoretical framework (Kovalenko et al., 2020). This is not the case with Mentors in Violence Prevention, which has strong underpinnings in social norms theory (Perkins & Berkowitz, 1986) and social cognitive models of decision-making (Ajzen, 1988, 1991; Gibbons & Gerrad, 1995, 1997). In addition, using the same sample and measures as this study, many socio-cognitive predictors have been shown to be significantly associated with intervention behavior (Pagani et al., 2022a). However, the extent to which these theoretical factors are explicitly addressed during lessons is less clear (Pagani et al., 2022b). Furthermore, how these factors are being framed within the context of the key messages that Mentors in Violence Prevention intends to communicate is unknown.

Strengths of the current study include the use of a longitudinal and multilevel design, and a robust theoretical framework, to examine intervention effects. However, the study was slightly underpowered to be able to detect meaningful effect sizes in some of the outcomes (namely, those pertaining to intervention behavior) as determined in the preregistered simulation analyses (OSF: 10.17605/OSF.IO/ZCJS6). Nonetheless, the design was sufficiently powered for all other anticipated outcomes, and the researchers endeavored to maintain consistency in the analyses used. Publication of null findings is an important step in building a comprehensive evidence base for this, or any, intervention. They inform future refinements in the application of interventions (Miller-Halegoua, 2017) and, since science cannot self-correct without them, they support progress more generally (Munafò & Neill, 2016). Second, although this study did include a multilevel component at the school level, it would have been useful to also include this at the classroom level to examine cross-classroom effects, where classroom culture could be captured. Indeed, other researchers have found larger effects at the classroom level than at the school level (Kärnä et al., 2011), suggesting that individuals belonging to the same classroom have more similar changes than those belonging to the same school. Unfortunately, the current study was neither statistically powered nor designed to include this further clustering step within the multilevel analysis. We were also unable to record the exact times at which implementation schools began and ended the intervention. This meant that the time between completing the intervention and undertaking the T2 assessments may have varied across schools, introducing undesirable variation in results. Furthermore, the fact that three of the Mentors in Violence Prevention schools became non-intervention schools due to their inability to implement the intervention, does raise questions about these schools’ capacity and willingness to implement the program, and could indicate a potential redistribution bias in the findings relating to non-intervention schools.

## Conclusion

Little is known about the effectiveness of bystander programmes targeting gender-based violence in the United Kingdom. Furthermore, studies tend to include a partial examination of bystander outcomes that do not fully comprise robust theoretical models of decision-making. The current study aimed to address these limitations by conducting a rigorous evaluation of the Mentors in Violence Prevention (Katz, 1995) program in Scottish high schools. Non-significant results revealed that Mentors in Violence Prevention was not found to influence changes after a 12-month follow-up in students’ self-reported intervention behaviors nor across any of the other anticipated outcomes, including attitudes, beliefs, motivations towards intervening, and willingness to intervene in less serious situations of gender-based violence. Multilevel modeling found that schools did not significantly differ in their changes, suggesting no between school variations in Mentors in Violence Prevention effects. Furthermore, exploratory analyses revealed that Mentors in Violence Prevention was not effective in positively influencing perceived behavioral control, intentions to intervene, and willingness to intervene in more serious situations of gender-based violence. This suggests that the intervention was not effective at modifying the anticipated outcomes in this study. The implications discussed indicate that decisions need to be made at stakeholder level as to whether Mentors in Violence Prevention should be framed according to its original intentions, that is, to tackle gender-based violence, and the likely implications of not doing so. The null results of the current study could further be attributed to a failure to adequately address the theoretical model underpinning Mentors in Violence Prevention in practice. These issues are reminiscent of wider issues in evaluating interventions, and should be addressed before concluding that an intervention is ineffective at bringing about its anticipated changes.

## Appendix 1

Table 1 Eight gender-based violence examples used from Miller et al. (2012)

**Table:**

Type of violence	Gender-based violence example
Verbal/emotional	A male peer/friend making rude or disrespectful comments about a girl’s body, clothes, or makeup.
	A male peer/friend doing unwelcome or uninvited things toward a girl (or group of girls), such as howling, whistling, or making sexual gestures.
	A male peer/friend spreading rumors about a girl’s sexual reputation, like saying she’s ‘easy to get with’.
	A male peer/friend telling sexual jokes that disrespect women and girls.
Physical/sexual	A male peer/friend showing other people sexual messages or naked/sexual pictures of a girl on a mobile phone or the internet.
	A male peer/friend arguing with a girl where he’s starting to swear at or threaten her.
	A male peer/friend shoving, grabbing, or otherwise physically hurting a girl.
	A male peer/friend taking sexual advantage of a girl (like touching, kissing, having sex with) who is drunk or high from drugs.

## Footnotes

1 Our preregistration on the Open Science Framework includes hypotheses, copies of all measures, relevant data, Mplus code, and other project materials such as participant consent and information sheets.

2 Single level linear regressions were also conducted on these outcomes, where the sample size was adequate to detect an effect. However, as was the case for the multilevel regressions (see results section), the linear regressions revealed that MVP had no significant effects on change scores for the more serious intervention outcomes (Negative intervention_MoreSerious_: β = –0.02, *p* = 0.847; Positive intervention_MoreSerious_: β = –0.19, *p* = 0.076).
